# DDT, epigenetic harm, and transgenerational environmental justice

**DOI:** 10.1186/1476-069X-13-62

**Published:** 2014-08-02

**Authors:** William P Kabasenche, Michael K Skinner

**Affiliations:** 1 Center for Reproductive Biology, School of Politics, Philosophy, and Public Affairs, Washington State University, Pullman, WA, USA; 2 Center for Reproductive Biology, School of Biological Sciences, Washington State University, Pullman, WA, USA

**Keywords:** DDT, Malaria, Africa, Bioethics, Generation, Inheritance, Environmental health

## Abstract

Although the environmentally harmful effects of widespread dichlorodiphenyltrichloroethane (DDT) use became well-known following Rachel Carson’s Silent Spring (1962), its human health effects have more recently become clearer. A ban on the use of DDT has been in place for over 30 years, but recently DDT has been used for malaria control in areas such as Africa. Recent work shows that DDT has transgenerational effects in progeny and generations never directly exposed to DDT. These effects have health implications for individuals who are not able to have any voice in the decision to use the pesticide. The transgenerational effects of DDT are considered in light of some widely accepted ethical principles. We argue that this reframes the decision to use DDT, requiring us to incorporate new considerations, and new kinds of decision making, into the deliberative process that determines its ongoing use. Ethical considerations for intergenerational environmental justice are presented that include concern and respect for autonomy, nonmaleficence, and justice. Here, we offer a characterization of the kinds of ethical considerations that must be taken into account in any satisfactory decisions to use DDT.

## Background

**Figure 1:**
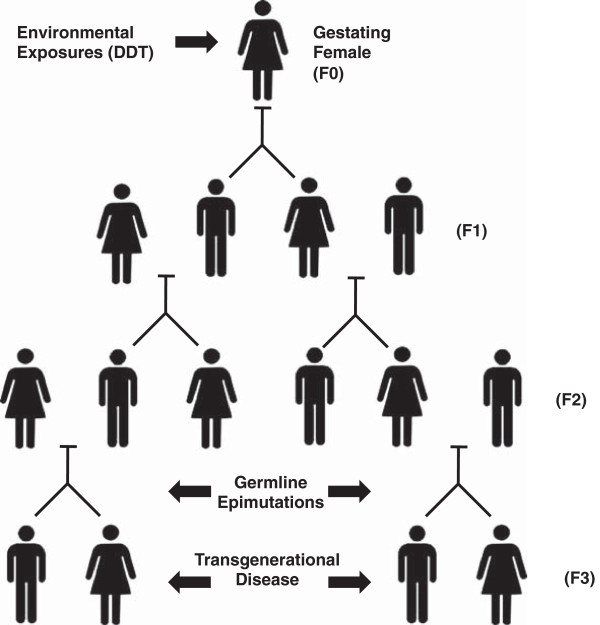
Scheme for DDT induced epigenetic transgenerational inheritance of disease.

A variety of environmental factors that include toxicants, nutrition and stress have been shown to induce the epigenetic transgenerational inheritance of disease [1,2] (Figure 1). Examples of such environmental compounds include pesticides [3,4], fungicide vinclozolin [3], hydrocarbons (jet fuel) [5], dioxin [6], and the plasticizers phthalates and bisphenol A (BPA) [7]. Nutritional effects such as high fat diets and caloric restriction can also promote transgenerational abnormalities [8]. Epigenetic transgenerational inheritance requires the germline (sperm or egg) transmission of epigenetic information that alters disease or phenotype, in the absence of direct environmental exposures [2]. Transgenerational phenomenon have been demonstrated in humans [9], rodents [3], worms [10], flies [11], and plants [12]. Therefore even though you have never had a direct exposure, your ancestors’ environmental exposures may influence your disease development (Figure 1). Environmentally induced epigenetic transgenerational inheritance of disease is a factor in disease etiology that needs to be considered in environmental policy.

A recent study examined the epigenetic transgenerational actions of the most common historically used insecticide DDT (dichlorodiphenyltoxichloroethane) [1]. Observations demonstrate that DDT has the ability to induce the epigenetic transgenerational inheritance of obesity, kidney, testis and ovary disease [1]. Although the United States and most developed countries have banned the use of DDT, recently it has been used globally as an insecticide for control of vectors for malaria. In 2001 the Stockholm Convention of United Nations Environmental Program proposed the elimination of 12 chemicals that induced DDT [13]. However, due to the recent Gates Foundation Malaria Control Program the use of DDT in Africa and other parts of the world has increased since the Stockholm Convention [14]. The World Health Organization (WHO) issued a position statement in 2006 promoting the use of indoor residual spraying with DDT for malaria vector control. The reported use of DDT globally for disease vector control is over 5,000 metric tons per year with India being the largest consumer [15]. Studies have indicated indoor spraying of DDT causes high levels of human exposure [16]. The direct DDT exposure toxic effects in humans include developmental abnormalities [17], reproductive disease [18], neurological disease [19], and cancer [20]. The exposure DDT metabolite DDE (dichlorodiphenyldichloroehtane) also promotes abnormal human health effects such as childhood diabetes and obesity [21]. Therefore, DDT exposure directly impacts human health [22]. DDT exposure also influences the health and promotes birth defects in wildlife [23]. Despite DDT being a low-cost anti-malaria tool, the adverse human health and environmental effects (e.g. extremely long half-life) of DDT use must be carefully weighed against the benefits of malaria control [24].

The book ‘Silent Spring’ by Rachel Carson was published over 50 years ago and revealed the hazards of DDT to human and wildlife health [25]. Currently the World Health Organization (WHO) and the Gates Foundation promote the use of DDT in developing countries in Africa for malaria control. The current day potential hazards of DDT exposures need to now be considered in light of the transgenerational actions of DDT [1]. The various transgenerational diseases promoted by DDT include obesity, kidney disease and ovarian disease [1]. The long-term health and economic effects on survivors [26] and subsequent generations [1] now needs to be considered with respect to the number of lives saved from malaria. A more careful risk-benefit consideration of the use of DDT is needed since other options exist with less toxic shorter half-life pesticides. The primary objective of the following discussion is to incorporate the concept of transgenerational inheritance.

## Discussion

The unique aspect of the emerging work on the epigenetic effects of DDT is that we now have good reason to believe that DDT will negatively affect future generations. This raises questions of *intergenerational environmental justice*. Environmental justice concerns the distribution of burdens and benefits on individuals via practices that affect our environment. In her work, Kristin Shrader-Frechette identifies the focus of *environmental* justice as being on the disproportionate burdens faced by socially disempowered individuals and groups (e.g., the poor and racial and ethnic minorities) [27,28]. There are now many accounts of these individuals and groups suffering the ill effects of environmental degradation. DDT use in the developing world looks set to be yet another case in that sad history. Some evidence suggests that the current generation is harmed by exposure to DDT. The recent work cited above indicates health hazards for descendants of those exposed now. Thus, the harm will only fully emerge over the course of a number of generations. This is why DDT use is also an issue of *intergenerational* justice.

Consideration of intergenerational justice invites us to examine how our practices and activities will impose burdens (and benefits) on those who will inhabit the world 50 or 100 or 500 years from now [29]. We now have good reason to believe, based on the evidence discussed above, that the use of DDT will impose burdens on individuals in the next two or four generations, at least, while the current generation enjoys the benefits of its use. As we discuss below, questions of intergenerational justice differ from other kinds of decisions. All the affected parties are not known in advance because some do not yet exist. Who comes to exist in the future, and what health deficits they might face, is determined by decisions, both individual and policy-level, made today. Of course, the question of what the present generation owes future generations is greatly complicated by the non-identity problem and related issues [30]. We will directly address these complications in future work, though here we hope to limit our claims so as to avoid the most difficult questions raised by that problem.

We characterize the ethical issues in terms of environmental injustice because those who might live in the future are the ultimate socially disempowered group. They can have no input into or control over environmental conditions that will affect their well-being. They are vulnerable to harms and have no clear opportunity to benefit from the current generation’s use of DDT.

The provisional case that current DDT use’s impacts on future generations is an instance of intergenerational environmental injustice can be developed in terms of three moral concerns. First, the offspring of those exposed to high levels of DDT today are harmed in that the offspring’s health interests are set back by ancestral exposure. The principle of *nonmaleficence* concisely expresses the widely-held moral conviction that it is wrong to harm another, other things being equal. Assuming that *any* offspring of individuals exposed to DDT will be harmed by the ancestral exposure, the principle of nonmaleficence applies, even to future generations. Second, while many individuals might consent to undergo risk or actual harm, for some compensating benefit, the offspring cannot consent prior to the onset of the mechanism of injury. This violates *respect for autonomy*, which would otherwise be expressed, partially, in the ability to make an informed consent to assume risk or harm. Of course, those who do not exist yet do not have any autonomy to respect. Thus, they cannot consent to take on the epigenetic harm that will affect whoever comes to exist. Finally, the principle of *justice* calls for the distribution of benefits and burdens (including harms) in some kind of principled manner. DDT use affecting future generations through epigenetic harm seems to be a good provisional example of an unfair imposition of harm without corresponding benefit. At the very least, justice would seem to require that anyone likely to be harmed by action taken today be able to have a “place at the table” in discussion of whether to use substances like DDT. The three principles discussed here are elaborated and defended in Beauchamp & Childress [31] (Table 1).

**Table 1:** Ethical considerations for intergenerational environmental justice

1	*Consent/Respect for Autonomy*: Members of future generations cannot consent to risks and harms imposed by earlier generations.
2	*Nonmaleficence*: Members of future generations are harmed, via health deficits associated with epigenetics, due to exposure of ancestors to DDT (and other toxicants).
3	*Justice*: Members of future generations bear a disproportionate balance of risks and harms, whereas members of the current generation, when DDT is being used, enjoy disproportionate benefits.

One objection might say that if DDT had not been used in the current generation (F0), then members of a future generation (F3) who are the progeny of F0 might not have come to exist (Figure 1). Members of the F0 generation might have died of malaria before having children. Thus, the alleged cause of harm to the F3 generation, the use of DDT in F0, might actually also be part of what enables F3 to come to exist. How might this affect the provisional claim that current DDT use (in F0) is ethically suspect? First, that the objection exists does not immediately justify the status quo. The objection is based on quite a few conditional claims. For example, if members of F3 never came to exist, they would not be harmed by not existing [30]. The non-identity problem raises notorious complex questions of why it would be wrong to bring into existence a person who suffers health deficits, but who would not exist if not for the mechanism that also caused those deficits. Here we lack the space to fully address this concern, but in future work we hope to develop an agent-based account of wrong action that can be used to address the counterintuitive implications of the non-identity problem. Wasserman argues that an agent’s reasons for acting can be the target of ethical evaluation [32]. Agents who act from moral vice or the absence of virtue might be ethically criticized even given the non-identity problem. We hope to develop this agent-based approach for dealing with actions that have transgenerational implications. While it is not clear to us that the current use of DDT is obviously wrong, it now requires, we think, a more elaborate justification given its epigenetic effects.

That deaths of members of F0 are avoidable, via malaria prevention, does raise an ethical concern in itself (again, the principle of nonmaleficence would be relevant here). But if there are alternative ways to prevent malaria deaths in F0, we should obviously consider them. A number of organochlorine pesticides with shorter half-lives (i.e. methoxychlor, aldrin, dieldrine and eldrin) have been used and shown not to be as persistent environmental contaminants [33]. More recently developed pesticides such as bifenthrin [34], chlorfenapyr [35], and pirimiphos [36] have been shown to be effective as alternatives for DDT consideration. Although the alternatives like methoxychlor may promote transgenerational disease [37], more recently developed pesticides such as nicotinoids are also alternatives to consider [38,39]. Clearly factors such as cost and half-life which would require more frequent distribution are factors, this consideration would have to be part of the decision making process [15]. However, as our understanding of the health deficits to future generations due to the current generation’s use of DDT become clearer, this can significantly shift the balance of burdens. The “true cost” of using the less expensive and long half-life pesticide is shifted to members of the F3 generation who experience that cost in terms of health deficits and in the money needed, if possible, to correct or ameliorate those health deficits. Their lives and well-being cannot be discounted in the same way that economists discount future commodities [40,41]. Thus, any future health care costs caused by actions taken today need to be incorporated into a cost-benefit assessment. We do not claim to have worked out that decision making process, but we do argue that new concerns about epigenetic harm and transgenerational inheritance should reframe that process. Policy makers need to incorporate these considerations of transgenerational justice into their deliberation.

Concern about the well-being of members of the F0 generation, as well as members of the F3 generation, appear to call for some sort of trade-off or balancing of benefits and burdens. While we do not have space here to fully consider all the ramifications of this trade-off, we believe it is important to recognize that the decision to use DDT in the current generation has this implication. Very briefly, we note that the F0 generation might benefit from DDT use by the preservation of life and health (freedom from malaria) in the current generation. The F0 generation might also experience some burdens associated with its exposure to DDT [42]. And, F0 might experience harms if DDT is not used. However, the F3 generation would not be harmed by not using DDT regardless of whether not using DDT harmed the F0 generation. In a scenario where members of the F3 generation never come to exist because their great-grandparents died prior to reproducing, there can be no harm to those who do not yet, or never, come to exist. Members of F3 would be harmed, again by appeal to the recent epigenetic findings, if DDT is used. Finally, it strikes us as important that a mechanism that might allow one to live (DDT use) would also be a mechanism that causes one’s health deficits. The ethics of reproducing is surely complicated, but, again, it is not clear that ensuring F3’s existence by means of inducing harm in those who come to exist is an obviously right action.

## Conclusions

On this admittedly brief analysis of the trade-offs, it is certainly not clear that the F3 generation would benefit more from current use of DDT than from not using it. If there are alternatives for preventing malaria in the F0 generation that do not cause epigenetic harm, then the case for using them would seem to be ethically superior to any trade-off scenario involving continued use of DDT.

We draw two conclusions from this analysis. First, because recent empirical findings show that DDT is likely to cause intergenerational harm, policies involving its use should be re-considered to incorporate these new concerns into the decision procedure to use DDT. We have tried to highlight some of those new concerns in ethical terms (Table 1). Second, the provisional case against DDT use is fairly strong. This further strengthens the call for alternative means of preventing malaria and for discontinuing DDT use. But even if we lack a conclusive argument against current DDT use, we believe we have done enough to shift the burden of proof back to the advocates of its use. Perhaps our most important conclusion is that an unreflective continuation of the status quo with respect to DDT use is unacceptable. It needs to be defended against concerns about the intergenerational effects it will cause.

## Abbreviations

BPA: Bisphenol A; DDE: Dichlorodiphenyldichloroehtane; DDT: Dichlorodiphenyltrichloroethane; F0: Generation pregnant female; F1: Generation fetus that becomes the offspring or children; F2: Generation (grandchildren); F3: Generation (great-grandchildren); WHO: The World Health Organization.

## Competing interests

The authors declare no competing financial interests.

## Authors’ contributions

MKS conceived the study. MKS and WPK designed and wrote the study. Both authors edited and approved the manuscript.
